# Impact of COVID-19 on the Mental Health of Healthcare Professionals in Pakistan

**DOI:** 10.7759/cureus.8974

**Published:** 2020-07-02

**Authors:** Ram Sandesh, Wajeeha Shahid, Kapeel Dev, Nikeeta Mandhan, Prem Shankar, Anam Shaikh, Amber Rizwan

**Affiliations:** 1 Medicine, Liaquat University of Medical and Health Sciences, Jamshoro, PAK; 2 Internal Medicine, Jinnah Sindh Medical University, Karachi, PAK; 3 Internal Medicine, Ghulam Muhammad Mahar Medical College, Sukkur, PAK; 4 Internal Medicine, Dr. Ruth K. M. Pfau Civil Hospital, Karachi, PAK; 5 Internal Medicine, Dow Medical College, Karachi, PAK; 6 Internal Medicine, Dow University of Health Sciences, Karachi, PAK; 7 Family Medicine, Dr. Ruth Pfau Hospital, Karachi, PAK

**Keywords:** covid 19, pakistan, mental health

## Abstract

Introduction

As a result of the ongoing COVID-19 pandemic, health care professionals (HDPs) are facing immense strain due to the heavy load of cases. In many cases, they work increasingly long hours, often with limited resources and a dubious infrastructure. Thus, it is important to check on the mental health of caregivers.

Methods and materials

This cross-sectional study was conducted in May 2020, at various hospitals in Karachi, Pakistan. All HCPs posted in the COVID-19 isolation wards were invited to participate and a total of 112 completed this study. A carefully structured form was created, which included the Depression Anxiety Stress Scale-21 (DASS-21).

Results

The overall mean score of anxiety was 19.01 ± 9.2, depression was 18.12 ± 10, and stress was 20.12 ± 12.0. There were 81 (72.3%) participants who suffered from moderate to extremely severe depression, 96 ( 85.7%) participants who suffered from moderate to extremely severe anxiety, and 101 (90.1%) participants who reported moderate to extreme stress levels

Conclusions

It is evident that there are a high number of healthcare workers affected by various psychological ailments such as anxiety, stress, and depression. It is important that the government take steps to ensure that HCPs' mental health is regularly checked and that efforts are made to reduce their burdens.

## Introduction

COVID-19, also known as coronavirus disease, was first identified in Wuhan, China, in late 2019. It is a highly contagious disease with a rapidly rising incidence globally. The World Health Organisation (WHO) recognized it as a pandemic due to its widespread transmission [1]. As is the standard procedure in such pandemics, a lockdown is usually enforced to limit the spread of the disease and reduce potentially new cases by maintaining social distancing in all public places [2]. The general public undertakes such safety measures, but health care professionals (HCPs) are unfortunately left exposed to deal with the many issues that arise due to this situation. Firstly, due to a huge load of cases as a result of the pandemic, health care professionals face increasingly long work hours, often with limited resources and a dubious infrastructure [3]. Secondly, they face physical discomfort and sometimes even breathing difficulties while wearing personal protective equipment (PPE), which is essential to keep them safe from exposure to the virus [4]. Another big problem faced by the HCPs is that because little is known about the new virus/disease, and therefore, no established protocols or evidence-based clinical treatments are prevalent, many HCPs feel unprepared to carry out their duties [4]. And then there is a very valid fear of autoinoculation and the risk of spreading the virus to their family and friends [5-6]. This fear leads HCPs to isolate themselves from their families, alter their daily routines, and even reduce their social support system, all in the hopes of keeping everybody potentially safe from themselves [4]. 

Unsurprisingly, all these factors take a toll on the mental health of HCPs. In 2003, during the outbreak of severe acute respiratory syndrome (SARS), 18-57% of HCPs suffered severe emotional problems and psychiatric symptoms both during and after the breakout [7]. In 2015, when the Middle East respiratory syndrome (MERS) emerged, also caused by a coronavirus, HCPs suffered dysphoria and stress [7]. Many other studies have reported that mental health implications for professionals involved in healthcare during epidemics and pandemics are long-lasting. Even after some time had transpired after such events, high levels of stress, anxiety, depression, and even post-traumatic stress disorder (PTSD) were observed in many cases [8-9].

Therefore, it is extremely important to identify the HCPs who are at high risk of burnout and are more likely to suffer from anxiety, depression and stress in this pandemic, so that help can be provided where and when needed. It is also equally important to identify and address the factors responsible for this stress. In this study, we will establish the frequency of HCPs affected by anxiety, depression, and stress, as well as determine the causative factors behind them. 

## Materials and methods

This cross-sectional study was conducted in May 2020, at various hospitals of Karachi, Pakistan. All health care professionals (HCPs) posted in the isolation wards for COVID-19 were invited to participate. A total of 112 HCPs completed this study. A carefully structured form was created, which included the English version of the Depression Anxiety Stress Scale - 21 (DASS-21). DASS-21 is a 21-item self-report validated tool designed to measure the three related negative emotional states, which are: depression, anxiety, and stress [10]. The online form also included various reasons that HCPs thought were predisposing factors for anxiety, stress, and depression. The participants could choose multiple reasons as applicable to themselves. The collected data were analyzed using SPSS Version 21.0 (IBM Corp, Armonk, NJ). Mean and standard deviation (SD) were calculated for the scores of anxiety, stress, and depression. Frequencies and percentages were calculated for the severity of DASS-21 and reasons predisposing healthcare professionals to anxiety, stress, and depression.

## Results

Out of the 112 health care professionals (HCPs) who participated in this study, 64 (57.1%) were male, and 48 (42.9%) were female. The overall mean score of anxiety was 19.01 ± 9.2, depression was 18.12 ± 10, and stress was 20.12 ± 12.0. There were 81 (72.3%) participants who suffered from moderate to extremely severe depression, 96 ( 85.7%) participants who suffered from moderate to extremely severe anxiety, and 101 (90.1%) participants who reported moderate to extreme stress levels (Table 1).

**Table 1:** Frequency of Anxiety, Depression and Stress among Health Care Providers Abbreviations, DASS-21: Depression Anxiety Stress Scale

DASS-21	Depression		Anxiety		Stress	
	Male (n=64)	Female (n=48)	Male (n=64)	Female (n=48)	Male (n=64)	Female (n=48)
Normal	6 (9.3%)	5 (10.4%)	0	5 (10.4%)	2 (3.1%)	2 (4.1%)
Mild	8 (12.5%)	12 (25%)	3 (4.6%)	8 (16.6%)	4 (6.2%)	3 (6.2%)
Moderate	16 (25%)	7 (14.5%)	15 (23.4%)	10 (20.8%)	16 (25%)	13 (27%)
Severe	25 (39%)	20 (41.6%)	32 (50%)	17 (35.4%)	29 (45.3%)	22 (45.8%)
Extremely Severe	9 (14%)	4 (8.3%)	14 (21.8%)	8 (16.6%)	13 (20.3%)	8 (16.6%)

The study found that the most common reason for stress and anxiety among HCPs treating COVID-19 positive patients was the fear that they might infect their family members (89.2%), followed by the fear of getting infected themselves (80.3%). Other reasons were: increased workload (64.2%), lack of PPE (62.5%), lack of security (62.5%), and lack of awareness among the general population about COVID-19 (46.4%) (Table 2).

**Table 2:** Reasons for Stress, Anxiety and Depression among Health Care Providers Abbreviations, PPE: Personal Protection Equipments

Reasons	Male (n=64)	Female (n=48)
Possibility of contracting COVID-19	51 (79.6%)	39 (81.2%)
Possibility of infecting their family	59 (92.2%)	41 (85.4%)
Lack of PPE	42 (65.6%)	28 (58.3%)
Lack of security	41 (64%)	29 (60.4%)
Increased workload	41 (64%)	31 (64.5%)
Lack of awareness among general population	30 (46.8%)	22 (45.8%)

## Discussion

The psychological response of HCPs to an epidemic could be dependent on many factors which may cause anxiety and stress such as feeling vulnerable to infection, lack of control over the situation, the spread of the virus, health of their family, and being isolated [11]. Other factors may also contribute to stress and anxiety among HCPs such as shortage of PPEs, medicines, etc. and an increasing number of actual and suspected cases [12]. These factors may contribute to various levels and severity of psychological pressure, which may lead to a feeling of loneliness and helplessness and may result in stress, anxiety, irritability, mental fatigue, and depression [4].

In this study, a significant number of Pakistani participants had anxiety, stress, and depression. Around 89% of HCPs were afraid for their family, and 80% of HCPs feared that they might get COVID-19 themselves. These figures can be compared to Wuhan, where among HCPs looking after COVID-19 patients, only 50.4% had depression, 44.6% had anxiety, 34% had insomnia, and a considerable proportion of participants had symptoms of depression (634 [50.4%]), anxiety (560 [44.6%]), insomnia (427 [34.0%]), and distress (899 [71.5%]) [13].

Factors such as improper infrastructure for patient care, lack of awareness among the masses, and poor compliance with safety measures can be accredited to this high prevalence of anxiety, depression, and stress in Pakistan. Another important thing to note is the persistence of mental health implications for healthcare workers. Even after some time had passed after an epidemic, many studies reported that high levels of anxiety, depression, stress, and even PTSD were observed in many HCPs [8]. 

In addition, the HCPs who are directly involved in the treatment of a highly contagious disease such as the COVID-19 may suffer a stigma whereas, at the other end of the spectrum, this pandemic has led to HCPs being given the 'superhero' status. On the one hand, this adds value and gratification to the job, but on the other, it puts more pressure on the workers, leaving no margin for error. Due to the sensational character of this worldwide pandemic, the 'superhero' status is being reinforced by the media, promoting the need for emotional support, encouragement, and appreciation [14]. 

In light of the results of this study, it is clear that an alarmingly high number of healthcare workers are the victims of mental health ill-effects caused by the COVID-19 pandemic. It can be also seen that many of the factors due to which HCPs suffer from these ill-effects can be potentially modified, for example, such as free provision of PPEs to all healthcare workers, promoting general public awareness about COVID-19, and building better infrastructure to encourage lighter work hours can all be carried out by the government and thus have a positive impact on the mental health of HCPs.

## Conclusions

In our study, high levels of anxiety, stress and depression among health care professionals were noted, which is a cause for concern. Both the government and health care agencies are responsible for protecting the psychological well-being of health care communities all over the world and ensuring a healthy work environment. Since there is a high prevalence of anxiety, depression, and stress among HCPs treating COVID-19 patients, it is imperative to invest resources to promote the mental health welfare of frontline professionals.
